# Writing Wrongs: Navigating Microaggressions with Adapted Expressive Writing for Minoritized College Students

**DOI:** 10.1007/s40615-025-02560-0

**Published:** 2025-08-01

**Authors:** Cassidy Brydon Chesnutt, Sydney N. Waitz-Kudla, Marielle Gomez, Cristina M. López, Tracy K. Witte

**Affiliations:** 1 Department of Psychological Sciences, Auburn University, Auburn, USA; 2 Department of Psychiatry & Behavioral Sciences, Medical University of South Carolina, Charleston, USA

**Keywords:** Expressive writing, Adaptation, Microaggression, Minoritized students

## Abstract

In addition to stressors inherent to higher education, microaggressions are pervasive and result in lasting negative repercussions for minoritized students at predominantly White institutions (PWIs). Expressive writing may be a scalable intervention fit for addressing these negative repercussions due to its established efficacy in improving symptoms related to general stressful life events. As expressive writing was not designed to address microaggressions, we utilized the ADAPT-ITT model to develop Writing Wrongs, a scalable intervention aimed at addressing microaggressions experienced by minoritized students at PWIs. The current study aimed to (a) assess attrition and pilot measures for future studies, (b) elicit impressions about Writing Wrongs’ necessity, (c) elicit feedback regarding Writing Wrongs, and (d) identify recommended changes to the intervention. We conducted a theater test with both standardized expressive writing and adapted Writing Wrongs prompts in a sample of 18 predominantly female racial/ethnic minoritized college students enrolled at a PWI. Utilizing a mixed-methods design, we found full retention following the first pre-intervention session; adequate variability for most quantitative measures; and that participants broadly perceived Writing Wrongs to be helpful, appropriate, appealing, and necessary per quantitative and qualitative feedback. Additionally, participants expressed a preference for the Writing Wrongs prompt over the standardized prompt in qualitative and quantitative feedback. Participants provided additional recommendations for future modifications of the prompt and administration of Writing Wrongs. The results of the current study can inform the next phases of the ADAPT-ITT model, with the intent of creating a finalized adaptation of Writing Wrongs.

**Supplementary Information:**

The online version contains supplementary material available at https://doi.org/10.1007/s40615-025-02560-0.

## Introduction

As of 2023, 48.9% of university students within the USA identify with a minoritized racial or ethnic group [1]. Along with the current climate and universally experienced academic stressors, minoritized students experience racial or ethnic stressors, such as microaggressions on campus [2, 3]. Microaggressions are daily experiences of indirect, subtle, or unintentional expressions of degradation, invalidation, or discrimination, which range from racial jokes to racial slurs [4, 5] and contribute to adverse mental, physical, and academic outcomes [5–8]. Experiences of microaggressions and associated negative repercussions tend to be exacerbated at predominantly White institutions (PWIs) as evidenced by findings from mixed methods studies that minoritized students experience frequent, severe, and wide-ranging microaggressions while attending PWIs [9, 10]. In addition to the increased frequency and severity of microaggressions experienced, there are few acceptable coping resources for discriminatory experiences [11]. Furthermore, many universities have been compelled to dismantle diversity, equity, and inclusion (DEI) offices and affiliated spaces (e.g., multicultural centers; pride centers) in response to recent federal and state legislative mandates restricting the funding of such initiatives. In this climate, expressive writing may be an effective and free coping resource to address the experience of microaggressions for minoritized students as it demonstrates efficacy in improving coping with general life stressors [12–15]. The goal of the proposed study is to promote well-being in minoritized students attending PWIs by developing Writing Wrongs, a scalable intervention designed to facilitate coping with microaggressions through expressive writing.

### ADAPT-ITT Model

An intervention for addressing microaggressions in a minoritized student population must be adapted beyond simply exporting an established intervention without modification to a minoritized population with unique presenting concerns. Though efficacious in a majority population for general stressors, interventions targeting the unique experiences of minoritized students warrant further consideration and feedback to ensure the necessity, acceptability, appropriateness, and (ultimately) efficacy of the intervention. The ADAPT-ITT model [16], an eight-phase standardized procedure for adopting or adapting an intervention for new populations, has demonstrated efficacy in adapting interventions to new groups and topics. The current study addressed the first six phases of the ADAPT-ITT model.

#### Assessment—Microaggressions and Minoritized Students at PWIs

Assessment, the first phase of the ADAPT-ITT model, is used to identify needs and potential solutions [16]. The current study is particularly interested in experiences of microaggressions, as the ambiguity and invisibility of microaggressions often lead to dismissal of potential harm [5]. Addressing microaggressions through direct intervention with aggressors, psychoeducation on the harm of microaggressions, or increasing availability of coping resources is further impacted by the perceived ambiguity, perceived invisibility, and ease of dismissal of microaggressions. Outcomes for minoritized students at PWIs who experience microaggressions include alterations to brain structures [17, 18], developing and worsening posttraumatic stress and depression symptoms [19, 20], increased negative affect [20], increased fatigue [5, 8], and increased self-monitoring and justification of identity [6]. Inconsistent with academic success, the compounding experiences of microaggressions are associated with a decreased sense of belonging [21] and higher rates of dropout and transfer [6, 7]. In sum, microaggressions experienced by minoritized students result in unique, compounding negative health repercussions not experienced by their White counterparts. It is important to note that addressing coping with microaggressions among minoritized students should not be viewed as the ultimate solution to this problem plaguing college campuses. Institution-level changes are also needed to prevent non-minoritized individuals from perpetrating microaggressions and other acts of racism from occurring in the first place. However, as these efforts unfold, an intervention that promotes coping among minoritized students is a worthy endeavor.

#### Decision—Potential Interventions

The second phase of the ADAPT-ITT model, decision, identifies an intervention that may be an appropriate solution to the previously identified need [16]. To our knowledge, there are no evidence-based interventions aimed at addressing microaggressions experienced by minoritized students in PWIs. Most interventions designed or adapted for minoritized students are either focused on more severe distress, like general or racially based traumas, and/or require a trained clinician for administration [22, 23]. Research points to significantly lower rates of treatment-seeking and mental health resource utilization in minoritized students compared to their White peers [11]. This trend may be attributable to pragmatic barriers (i.e., money, time, availability, transportation) [11, 24] or to treatment barriers, such as historically based mistrust of the clinical environment [25, 26] worsened by a lack of representation, cultural competence, and cultural humility among clinicians [27]. A potential solution to such barriers is the use of scalable interventions. Scalable interventions are brief, non-specialist-delivered, self-help-based, accessible, and/or easily distributed [28–30]. Currently available scalable interventions target general stressors, general traumas, or traumas unique to other minoritized groups and may take the form of mobile apps or a writing technique [31–33]. While previous work on a culturally responsive scalable intervention designed for African American students showed efficacy [33], it did not specifically address the negative repercussions of microaggressions. An intervention with much research promise that can be both scalable and adapted to address microaggressions experienced by minoritized students at PWIs is expressive writing [13, 31, 32].

##### Expressive Writing

Expressive writing is a brief intervention developed by Pennebaker and Beall [13] that has been operationalized as a type of repeated exposure practice that asks individuals to expressively write about their thoughts and feelings regarding a stressful life event. Consistent with general exposure practices, initial increases in negative affect are often observed in the first session of expressive writing [12, 14, 15]. After completing expressive writing, general benefits established by previous research include, but are not limited to, the development of improved coping strategies, reduction of rumination symptoms, improved emotion regulation, and reduction of activity restriction [12–15]. In minoritized students, specifically, expressive writing demonstrates similar benefits when writing about general stress and trauma [31, 34]. Despite the established benefits of expressive writing, critiques of the intervention call into question its efficacy in addressing posttraumatic stress symptoms related to the *Diagnostic and Statistical Manual, Fifth Edition* (DSM-5) criterion A traumas, specifically, and in comparison to clinician-administered trauma-focused interventions (i.e., prolonged exposure, written exposure therapy) [12, 14, 15, 35]. These critics also acknowledge expressive writing’s ability to reduce symptoms related to general life stressors that do not meet DSM-5 criterion A requirements [12, 14, 15, 35]. Expressive writing’s critiques lend further support for expressive writing’s potential utility in addressing microaggressions, a general life stressor that does not meet DSM-5 criterion A requirements.

The traditional administration of expressive writing includes three 20-min writing sessions across consecutive days within a clinical setting, where the prompt asks participants to write about a stressful life event and feelings associated with the event [14, 15, 36]. Expressive writing has also been administered in a laboratory setting, online, half in-person and half online, on paper, or on computers [31, 37]. Minoritized student populations may benefit from a scalable online administration method of expressive writing, bypassing numerous barriers to treatment. When traditional expressive writing for traumatic experiences has been administered online, it has demonstrated efficacy across diverse populations including LGBTQ+ [12, 38], Asian [34], and Hispanic [31] populations.

Despite the evidence suggesting that microaggressions take a significant mental and physical health toll on minoritized students at PWIs and that expressive writing is an efficacious scalable intervention to address stressful life experiences that may generalize to microaggressions, it is still unknown how expressive writing would need to be adapted or adopted to target microaggressions experienced by this population.

### Current Study

In the current study, we aimed to address the next four phases of the ADAPT-ITT model: administration, production, topical experts, and integration. We addressed the Administration phase by conducting a theater test of expressive writing as a potential intervention for symptoms resulting from microaggressions experienced by minoritized students attending PWIs. As defined by Wingood and DiClemente [16], the goal of a theater test is to collect feedback on the materials, content, and delivery of an intervention from at least 15 individuals in the intended audience. The theater test included the unadapted and adapted prompt. The adapted prompt, titled Writing Wrongs, was developed in consultation with topical experts and the target population as an acceptability trial, addressing the production and topical experts phases (i.e., ADAPT-ITT steps 4 and 5). The title was specifically chosen to represent the opportunity for individuals to cope with microaggressions, or wrongs, through writing. The collection of various forms of feedback throughout the current study addressed the integration phase. Research aims for our study included the following: (a) investigating attrition rates across study timepoints and piloting questionnaires measuring the effects and frequency of microaggressions experienced, mental health symptoms before and after expressive writing, and acculturation status of our sample to establish their psychometric properties; (b) eliciting impressions about the desirability of expressive writing as an intervention for microaggressions experienced by minoritized students at PWIs; (c) eliciting feedback regarding the unadapted and adapted Writing Wrongs prompts; and (d) identifying recommended changes to the intervention by participants.

## Methods

### Participants and Procedure

**Fig. 1:**
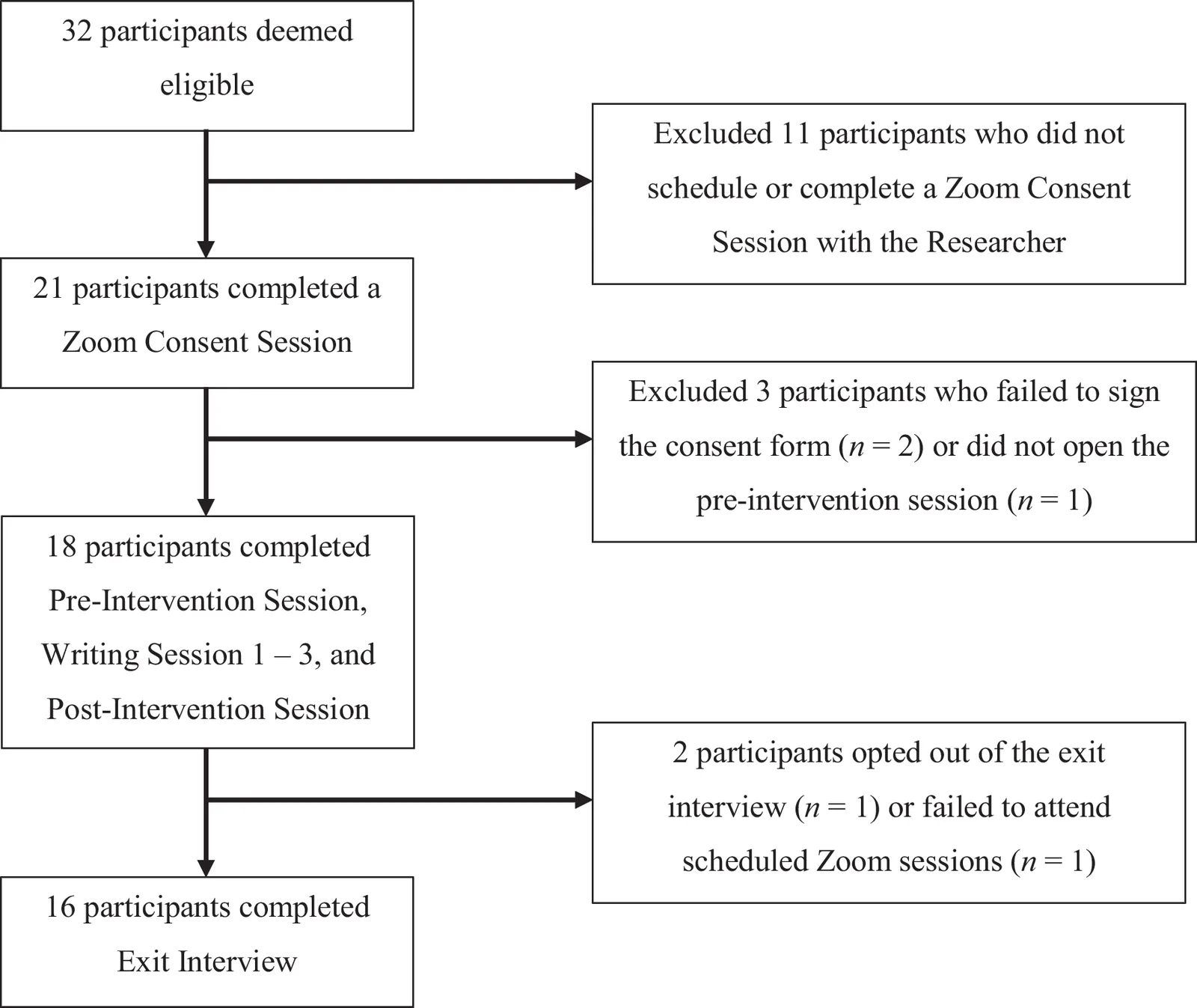
Study flow

Participants were required to be full-time students enrolled in a large PWI in the southeastern USA, be at least 18 years of age, and identify with a racial or ethnic minoritized group. Participants were recruited via flyers, social media posts, emails to relevant campus organizations, and university research participant pools. Although enrollment remained open to all who met inclusion criteria, advertising strategies were monitored and modified in an attempt to recruit a balanced sample (e.g., posting flyers in gyms, sending emails to specific organizations). Of the 32 students who responded to the study’s advertisements and met eligibility criteria, 18 participated in the writing intervention (Figure 1). Students who failed to complete the consent process by failing to respond to scheduling emails or log onto the Zoom consent call or failing to complete the consent form or start the pre-session received no further communication from study personnel. Demographics can be found in Table 1. The final sample was predominantly female, ranged in age from 18 to 29, and consisted of students who identified with a variety of racial and ethnic minoritized groups. All procedures were approved by the university’s Institutional Review Board.

**Table 1:** Sample characteristics (*N* = 18)

Demographics	Mean (SD)
Demographic	*n* (%)
Age	22.28 (3.83)
Gender	
Female	12 (66.7%)
Male	5 (27.8%)
None reported	1 (5.6%)
Race/ethnicity	
Black/African American^a^	6 (33.3%)
Asian^a^	5 (27.8%)
Hispanic/Latine^a^	4 (22.2%)
Multiple racial/ethnic groups identified	3 (16.7%)
Not listed (e.g., “Persian,” “African”)^b^	2 (11.1%)
Sexual orientation	
Straight/heterosexual	13 (72.2%)
Bisexual	3 (16.7%)
Pansexual	1 (5.6%)
Asexual	1 (5.6%)
Nationality	
US citizen	14 (77.8%)
Non-US citizen	4 (22.2%)
Student status	
Undergraduate	13 (72.2%)
First year	5 (27.8%)
Second year	3 (16.7%)
Fourth year	4 (22.2%)
Fifth year and beyond	1 (5.6%)
Graduate	5 (27.8%)

^a^Racial/ethnic identities are listed as they were presented to the participants

^b^These participants provided text responses to self-identify their race and ethnicity

The informed consent session was held via Zoom with a research assistant. On the intervention start date, the researcher sent the participants a pre-intervention Qualtrics survey. A list of measures administered at each time point is provided in Table S1 in the online supplemental materials. The rest of the study consisted of three 20-min writing sessions with pre- and post-writing questionnaires over consecutive days, the post-assessment session, and an optional exit interview. Writing Sessions 1 and 3 consisted of the standard expressive writing prompt [30]. Writing Session 2 consisted of the adapted Writing Wrongs prompt (Table 2). Participants also completed a post-intervention assessment after Writing Session 3 and indicated their interest in an optional Zoom exit interview. Participants indicated their preference between monetary and course credit compensation types and received up to $40 or 4.0 credits for completing all study sessions. Compensation was allocated across sessions at $5 or 0.5 credits for the first three sessions and $10 or 1.0 credits for the fourth session. Participants who opted into the exit interview (*n* = 16; 89%) received additional compensation ($10 or 1.0 credits), as well as a completion bonus ($5 or 0.5 credits) for engaging in all four sessions along with the exit interview.

**Table 2:** Writing Wrongs prompt

Writing Wrongs prompt
Please read the following instructions carefully as they have changed from your last session. You will have 20 minutes to write about a time that you experienced a **microaggression. Microaggressions are like papercuts. They are subtle everyday spoken, unspoken, or environmental interactions where someone expresses an insulting, degrading, or minimizing sentiment about you or a group with which you identify (e.g., race, ethnicity, gender, sexuality, etc.). Microaggressions can also be intentional or unintentional.** This experience should be something you experienced and not something witnessed. Please include both information on the **microaggression** and how it made you feel. Please also discuss how the **microaggression** impacted you emotionally both during and afterwards.

Strikethrough and bold font represent what was changed compared to the standard expressive writing prompt

### Measures

#### Demographics Information Questionnaire

Demographic information included can be found in Table 1.

##### Measures of Acculturation, Microaggressions, and Discrimination

See online supplemental materials Tables S3–S4 for descriptive statistics.

##### Vancouver Index of Acculturation (VIA; ***α*** = .86) [39]^1^

It is a 20-item scale that assesses the degree to which participants identify with and participate in the dominant and nondominant cultures. Items are rated on a 1 (*disagree*) to 9 (*agree*) scale, and there are subscale scores for heritage/nondominant culture and mainstream/dominant culture.

##### Racial Microaggression Scale (RMAS) [40]

It is a 46-item scale that measures how frequently individuals experience racial microaggressions and the distress they cause. The items are organized into six subscales according to the categories of microaggressions proposed by Sue et al. [5]: foreigner, criminality, sexualization, low achieving, invisibility, and environmental subscales. Each item is rated on both frequency (0 [*never*] to 3 [*often/frequently*]) and distress (0 [*not at all*] to 3 [*high level*]). Research supports the validity of the RMAS across multiple racial and ethnic minoritized populations [41]. In our sample, the measure demonstrated adequate internal consistency across several frequency subscales with alphas ranging from.73 to .89 for subscales aside from invisibility (.60) and environmental (.62). For the distress subscales, participants only provided ratings for items they identified as occurring at least *a little/rarely* (1). As such, we only calculated internal consistency for foreigner (i.e., .76) and environmental (.80).

##### Everyday Discrimination Scale (EDS; ***α*** = .81) [42]

It is a nine-item scale measuring the frequency of discriminatory events, rated on a frequency scale (1 = *never* to 7 = *always*). 

##### Clinical Outcome Measures

See Table 3 for descriptive statistics.

**Table 3:** Clinical outcome measures (*N* =18)

	Measure range	Observed range	Pre-session		Writing Session 1				Writing Session 2				Writing Session 3			
					Pre-writing		Post-writing		Pre-writing		Post-writing		Pre-writing		Post-writing	
			*M*	SD	*M*	SD	*M*	SD	*M*	SD	*M*	SD	*M*	SD	*M*	SD
PANAS																
Positive affect	10–50	12–48	-	-	29.3	8.6	24.3	9.6	20.4	7.8	25.2	10.2	23.0	9.6	22.4	10.3
Negative affect	10–50	10–34	-	-	15.7	16.1	18.8	6.4	14.3	5.7	14.2	5.5	14.1	5.5	16.1	6.4
Anxiety	0–8	0–6	-	-	-	-	2.0	1.8	-	-	0.9	1.6	-	-	1.3	1.7
Depression	0–8	0–8	-	-	-	-	3.1	2.4	-	-	1.6	1.6	-	-	2.3	2.4
SPRINT	0–32	1–23	-	-	-	-	11.8	6.4	-	-	7.3	5.9	-	-	9.7	6.7
DASS																
Depression	0–21	0–16	6.5	4.7	-	-	-	-	-	-	-	-	-	-	4.6	4.3
Anxiety	0–21	0–13	5.2	3.9	-	-	-	-	-	-	-	-	-	-	3.0	3.8
Stress	0–21	0–16	7.9	4.8	-	-	-	-	-	-	-	-	-	-	6.2	5.0
Total distress	0–63	0–42	19.6	11.7	-	-	-	-	-	-	-	-	-	-	13.8	12.2
PCL-5^a^	0–80	0–48	14.0	17.9	-	-	-	-	-	-	-	-	-	-	12.7	12.2

Positive and Negative Affect Scale (PANAS), Short Post-Traumatic Stress Disorder Rating Interview (SPRINT), Depression Anxiety and Stress Scale (DASS), Posttraumatic Stress Disorder Checklist – Diagnostic and Statistical Manual of Mental Disorders, Fifth Edition (PCL-5). Cells with a “-” reflect data that were not collected

^a^11 participants’ scores were removed as their traumatic event did not meet DSM-5 Criterion A. Reported statistics are based on *n* = 7

##### Positive and Negative Affect Scale (PANAS) [43]

For the purposes of this study, participants rated the 20 listed emotions based on how they were feeling at the present moment using a scale ranging from 1 (*very slightly or not at all*) to 5 (*extremely*). Scores were separated into positive and negative affect scales. The measure demonstrated adequate internal consistency in our sample, with alphas ranging from.71 to .90 for positive and .67 to.80 for negative affect across all writing sessions.

##### Depression, Anxiety, and Stress Scale (DASS-21) [44]

It is a 21-item scale that assesses symptoms of anxiety and depression experienced by the participant in the last week. Items are further divided into depression, anxiety, and stress subscales, with seven items each. Each item is rated on a 4-point scale ranging from 0 (*did not apply to me at all*) to 3 (*applied to me very much or most of the time*). To attain severity, scores on the subscales are totaled and multiplied by two. Higher scores indicate increased severity. In our sample, the measure demonstrated good internal consistency across the subscales, with alphas ranging from .84 to .88 for depression, .78 to .88 for anxiety, .85 to .88 for stress, and .92 to .95 for the total scale.

##### Life Events Checklist (LEC-5) [45]

It is a measure of trauma history based on 17 potential traumatic events. Each event is rated as *happened to you*, *witnessed it*, *learned about it*, *not sure*, or *does not apply*. Participants answer questions concerning their age when the event occurred, if the event happened more than once, if anyone was seriously injured, threatened, or killed, and if someone’s life was in danger.

##### Posttraumatic Stress Disorder Checklist – DSM 5 (PCL-5) [46]

It is a 20-item scale corresponding to the *DSM-5* symptoms for PTSD. Participants were asked to identify the worst event from the LEC-5 and respond to the PCL-5 based on this event. Participants answered on a 5-point scale how much each symptom has bothered them in the past month (0, *not at all*, to 4, *extremely*). To assess *DSM-5* PTSD Criterion A, the first author reviewed the participants’ narrative responses to the worst event from the LEC-5. Seven participants’ worst event met Criterion A per the *DSM-5.* Descriptive statistics for the PCL-5 were measured in this sub-sample, and the PCL-5 showed an adequate internal consistency with alphas ranging from .92 to .96.

##### Assessment of Anxiety and Depression [47]

It is a four-item measure with two items for ratings of anxiety and depression. Participants answered on a 5-point rating scale about how much they felt anxious, nervous, downhearted, and sad over the last 24 h (0, *not at all*, to 4, *very much*). The measure demonstrated good internal consistency in our sample with alphas ranging from .85 to .94 and .72 to .91 for anxiety and depression, respectively.

##### Short Post-Traumatic Stress Disorder Rating Interview (SPRINT) [48]

It is an eight-item measure that assesses posttraumatic stress symptoms related to the event the participant wrote about during the writing session. Participants rated how much each symptom has bothered them in the past week (0, *not at all*, to 4, e*xtremely*). The measure demonstrated good internal consistency in our sample, with alphas ranging from .81 to .85.

#### Intervention Feedback Measures

##### Adaptation Evaluation Form

This measure was adapted from Wingood and DiClemente’s [16] guide for the ADAPT-ITT model and was modified for each writing session to understand the acceptability, perceptions, and desired changes of the intervention. All Adaptation Evaluation Forms included quantitative and qualitative questions relating to participants’ opinions on how helpful and liked the writing prompt was and if they wrote about a racially/ethnically based stressor. The final Adaptation Evaluation Form following Writing Session 3 included additional questions regarding the participants’ opinions about the activity overall, the administration method (i.e., online format, device used), and what changes they would recommend. See tables for frequencies for quantitative questions (Table 4) and qualitative themes (Table 5) most central to our research aims. Additional qualitative themes (Table S5) may be found in the online supplemental materials.

**Table 4:** Acceptance and approval quantitative measures

Acceptance measures	Writing Session 1	Writing Session 2	Writing Session 3
	*n* (%)	*n* (%)	*n* (%)
How much do you like this activity as a whole?			
Not at all	-	-	1 (5.6)
Slightly	-	-	1 (5.6)
Somewhat	-	-	5 (27.8)
Quite a bit	-	-	7 (38.9)
Very much	-	-	4 (22.2)
How much did the activity feel relevant to your experience?			
Not at all	-	-	1 (5.6)
Slightly	-	-	2 (11.1)
Somewhat	-	-	3 (16.7)
Quite a bit	-	-	6 (33.3)
Very much	-	-	6 (33.3)
How much do you feel like this activity helped you?			
Not at all	-	-	2 (11.1)
Slightly	-	-	3 (16.7)
Somewhat	-	-	5 (27.8)
Quite a bit	-	-	3 (16.7)
Very much	-	-	4 (22.2)
How much do you feel like this writing activity helped you?			
Not at all	1 (5.6)	2 (11.1)	2 (11.1)
Slightly	5 (27.8)	1 (5.6)	3 (16.7)
Somewhat	7 (38.9)	6 (33.3)	6 (33.3)
Quite a bit	2 (11.1)	6 (33.3)	3 (16.7)
Very much	3 (16.7)	3 (16.7)	3 (16.7)
How much did you like how this session’s writing prompt was worded?			
Not at all	1 (5.6)	1 (5.6)	-
Slightly	1 (5.6)	3 (16.7)	-
Somewhat	5 (27.8)	4 (22.2)	-
Quite a bit	4 (27.8)	3 (16.7)	-
Very much	7 (38.9)	7 (38.9)	-
How fair do you feel the compensation was?			
Slightly	-	-	1 (5.6)
Somewhat	-	-	4 (22.2)
Quite a bit	-	-	3 (16.7)
Very much	-	-	10 (55.6)
How much did you like the online format of this activity?			
Somewhat	-	-	2 (11.1)
Quite a bit	-	-	5 (27.8)
Very much	-	-	11 (61.1)
How much did you like participating in the intervention with the device you used?			
Slightly	-	-	2 (11.1)
Somewhat	-	-	2 (11.1)
Quite a bit	-	-	3 (16.7)
Very much	-	-	11 (61.1)
How much is this an activity you’d like to do/want?			
Not at all	-	-	1 (5.6)
Slightly	-	-	6 (33.3)
Somewhat	-	-	2 (11.1)
Quite a bit	-	-	5 (27.8)
Very much	-	-	4 (22.2)

**Table 5:** Themes of responses to “How or why do you feel this activity did or did not help you?” at the end of each writing session (*N* = 18)

Theme and subtheme	Subtheme	Description	Writing Session 1	Writing Session 2	Writing Session 3
			*n* (%)	*n* (%)	*n* (%)
Emotional processing		Statements under this code relay that the intervention is “therapeutic” or aids in processing or providing clarity on emotions.	12 (67)	6 (33)	9 (50)
	Empowering/validating	Statements under this code indicate empowerment or validation coming from their engagement with the intervention.	2 (11)	1 (6)	2 (11)
Express experiences		Statements under this code indicate an ability to “put into words” the participants experience with the stressors.	5 (28)	5 (28)	4 (22)
	Gauge progress	Statements under this code reflect using the intervention to estimate the stage of processing or progress in managing distress related to the stressful event discussed.	2 (11)	0 (0)	2 (11)
Recall		Statements under this code indicate the intervention helped because it helped them recall an event/stressor.	2 (11)	2 (11)	1 (6)
Sharing experience		Statements under this code indicate the aspect of others reading their writing or knowing that the writing may be shared provided some benefit.	3 (17)	1 (6)	0 (0)
Education on microaggressions		Statements under this code indicate that learning what microaggressions are, improving identification of microaggressions, or creating a label for the experience of microaggressions made the intervention helpful.	0 (0)	2 (11)	0 (0)
Didn’t help		Statements under this code indicate that the intervention was not helpful.	1 (6)	3 (17)	2 (11)
Distressing but helpful		Statements under this code indicate that the activity was helpful in that it was valuable to write about the situation, but the experience provided more stress or distress than relief at this time.	0 (0)	1 (6)	1(6)

All 18 participants were administered this question; however, not all presentations of the question resulted in a response

##### Exit Interview Questionnaire

This interview was administered by the first author via Zoom to further understand the acceptability, perceptions, and utility of the intervention. The interviewer asked questions in a neutral manner and welcomed positive and negative feedback. The interviews were recorded and transcribed by trained undergraduate research assistants. See Table 6 for qualitative themes.

**Table 6:** Themes from spontaneously provided feedback during exit interviews (*n* = 16)

Themes and subthemes	Description	*n* (%)
General feedback	Statements under this code provide general feedback on the intervention as a whole.	16 (100)
Did help	Statements under this code indicate that the intervention was helpful.	15 (94)
Emotional processing	Statements under this code relay that the intervention is “therapeutic” or aids in processing or providing clarity on emotions.	12 (75)
Express experiences	Statements under this code indicate the intervention allowed participants an ability to “put into words” their experiences with the stressors through this intervention.	9 (56)
Distressed but helpful	Statements under this code indicate that the activity was helpful in that it was valuable to write about the situation, but the experience provided more stress or distress than relief at this time.	5 (31)
Education on microaggressions	Statements under this code indicate that learning what microaggressions are, improving identification of microaggressions, or creating a label for the experience of microaggressions made the intervention helpful.	2 (13)
Didn’t help	Statements under this code indicate that the intervention was not helpful.	3 (19)
Unpleasant	Statements under this code indicate aspects of the intervention that were unpleasant. Some participants identified unpleasant aspects of the intervention including thinking about the stressor during the writing activity.	6 (38)
Prompts	Statements under this code discuss the prompts.	16 (100)
Liked both	Statements under this code indicated that they liked both prompts.	4 (25)
Prompt 2	Statements under this code discuss prompt 2, the Writing Wrongs microaggression prompt.	13 (81)
Prompt 2 preference	Statements under this code indicate that the participant preferred prompt 2 or liked it more than prompt 1, the general prompt.	9 (56)
Microaggression definition	Statements under this code refer to the wording of the microaggressions prompt and requesting modification. For example, providing more detail or examples and removing the comparison to papercuts.	4 (25)
Prompt 1	Statements under this code discuss prompt 1, the general prompt.	12 (75)
More detailed prompt	Statements under this code requested more information, direction, or richer prompt descriptions. The statements may further indicate that prompt 1, the general prompt, is too broad.	9 (56)
Prompt 1 preference	Statements under this code indicate that the participant preferred prompt 1 or liked it more than prompt 2, the microaggression prompt.	3 (19)
Generally more specific	Statements under this code indicate a preference for more detailed prompts across both prompts (i.e., general prompt and microaggression specific prompt). For example, questions that ask more specifically about things such as stressors/microaggressions that are witnessed, experienced, parental, or generational.	8 (50)
New prompts each session	Statements under this code request new prompts each session.	6 (38)
Format	Statements under this code indicate that the participant discussed the format. This may include the online format, the personalized emails, or some other aspect.	16 (100)
Liked	Statements under this code indicate what participants liked the format.	10 (63)
Sessions	Statements under this code discussed a preference for the number of sessions or time between sessions.	16 (100)
Timeframe liked	Statements under this code preferred the 24 h between sessions.	10 (63)
Timeframe disliked	Statements under this code indicate that the “deadline” or timeframe of 24 h between sessions was disliked or unpleasant.	2 (13)
Measures disliked	Statements under this code indicate that the participant disliked the measures associated with the writing sessions. This may include the feedback or the PANAS.	8 (50)
Measures liked	Statements under this code indicated that the measures administered with the writing activity (i.e., the PANAS) was helpful.	4 (25)
Online	Statements under this code indicate a preference for the online format.	12 (75)
Text messages	Statements under this code discuss receiving the link over text message.	4 (25)
Text messages preferred	Statements under this code indicate a preference for the link to be sent via text message.	3 (19)
Hard to move to computer	Statements under this code indicate that receiving the link via text message was hard to translate over to another non-cellular device.	2 (13)
Additional resources	Statements under this code indicate a request for additional resources throughout the writing sessions.	4 (25)
Feedback	Statements under this code indicate a desire for feedback or the option for communicating with another individual about the writing.	4 (25)
Anonymity	Statements under this code indicate that the anonymity of the writing was beneficial or attractive.	1 (6)

### Data Analysis

To conduct our quantitative analyses, we first derived descriptive statistics of our sample, previously discussed. Due to the small sample size, we did not conduct inferential statistics. To analyze expressive writing texts, we used Pennebaker’s Linguistic Inquiry and Word Count-22 (LIWC-22) program, an online software used for analyzing word use in text entries [49]. Results were highly consistent with other measures (e.g., PANAS, qualitative and quantitative feedback) and are provided in the online supplemental materials *Description of Expressive Writing Text Analyses* section and in Tables S6–S7.

To conduct our qualitative analysis, we used thematic analysis to identify and analyze themes present in the expressive writing sessions, feedback assessments, and exit interviews [50]. The codes across the dataset were theory-driven and focused solely on comments relating to the primary study aims [50]. Two graduate student raters reviewed the qualitative data independently to identify broad themes aligned with the study aims. Once this was completed, the graduate students came together to discuss coding and resolve disagreements (e.g., conflicting code applications, refinement of code descriptions and names to better fit the theme). Upon resolution, the two graduate students generated a final list of themes and their applications within the dataset. Due to the consistent collaboration to ensure consensus, we did not compute Cohen’s *κ* statistic to calculate interrater reliability [51–53]. The primary investigator’s faculty mentor provided resolution of disagreements and refinement of the overall codebook. Dedoose™, an online software application used for qualitative data analysis, was used by the graduate students to assign codes to individual excerpts and to store code applications to inform the generation of a final codebook [54].

## Results

### Attrition and Completion

No attrition was observed following the completion of the pre-intervention session (Figure 1). Participants spent more time writing in response to the Writing Wrongs prompt (Session 2; median = 16.0 min), as compared to the general prompt (Sessions 1 [median = 14.8 minutes] and 3 [median = 12.9 min]). See online supplemental materials Table S2 for a descriptive table of timing for each session. Only one participant demonstrated a lack of engagement during one writing session (Session 3) by leaving the text box blank.

### Quantitative Measures of Acculturation and Discrimination

Participants appeared to be equally identified with the dominant (North American) culture as they were with the non-dominant (heritage) cultures on the VIA. When considering the experience of discrimination using the RMAS, participants rated environmentally based discrimination, not belonging/being treated as a foreigner, and low achieving as more frequently experienced forms of discrimination compared to the other subscales. When considering distress, participants indicated the most distress with discrimination relating to low achieving, invisibility, and criminality.

On the EDS, participants indicated experiencing discrimination relating to others being less courteous, less respectful, underestimating intelligence, and assuming superiority more frequently than other items on the scale. The most frequently endorsed identities targeted by the discriminatory experience were shade of skin color (*n* = 12; 66.7%), race (*n* = 11; 61.1%), ancestry or national origins (*n* = 8; 44.4%), and gender (*n* = 6; 33.3%).

### Clinical Outcome Measures

Clinical outcome measures demonstrated adequate variability in our sample. See Table 3 for descriptive statistics.

### Analyses of the Content Contained Within the Expressive Writing Text

As expected, qualitative analyses considering the expressive writing text of each participant found that identity-related stressors were discussed more often in response to the adapted Writing Wrongs prompt as compared to the general prompt (see online supplemental materials). These findings were further confirmed by the LIWC-22 ethnicity variables (see online supplemental materials). Furthermore, nearly all participants reported that they considered writing about an ethnically or racially based stressor for the Writing Wrongs prompt (*n* = 16), whereas only a minority of participants indicated that they considered such a stressor for Writing Sessions 1 (*n* = 4) and 3 (*n* = 6). Qualitatively, participants who did not write about an ethnically or racially based stressor during Writing Session 1, the general prompt, most frequently cited experiencing more salient stressors (*n* = 14). A majority of these participants (*n* = 9) indicated a sense of normalized discrimination (e.g., “I’ve just grown used to it,”) while others (*n* = 2) reported being unaware that writing about a racially or ethnically based stressor was an option due to the phrasing of the general prompt. The participants who did write about ethnically or racially based stressors in Writing Session 1 indicated that writing about identity was unintentional (*n* = 2; e.g., “just happened”), that their worldview was influenced by identity (*n* = 1), or that they suspected the study was related to ethnically or racially based topics (*n* = 1).

### Acceptance and Approval Across Quantitative and Qualitative Measures

When asked to consider the entire intervention, 94.5% (*n* = 16) of participants indicated they liked the intervention at least *somewhat*, and 83.3% (*n* = 15) of participants indicated that they found the intervention at least *somewhat* relevant to their experience (Table 4). Additionally, 66.7% (*n* = 12) of participants indicated that they found this intervention at least *somewhat* helpful (Table 4). Most participants also stated that the intervention was helpful in the exit interviews (Table 6). One participant reflected:

I liked it. It gave me time, ’cause sometimes life moves fast, so you’re trying to bottle things up or just push it to the side so it really gave me time to think. Especially about how it made me feel in certain moments and how I feel about it now. So, I liked it.

Consistent across both the exit interviews and the written feedback (Tables 5 and 6), participants reflected that emotional processing, empowerment, and validation; the opportunity to express experiences or put experiences into words; and the ability to recall the event or stressor were helpful aspects of the writing activity. Beyond this, some participants found it helpful to share the experience knowing that someone would read their reflections. A minority of participants (*n*_written feedback_ = 2, *n*_exit interview_ = 5) indicated that they found the intervention to be distressing but helpful. A small number of participants stated that the intervention did not help them (*n*_written feedback_ = 6, *n*_exit interview_ = 3) or was unpleasant (*n*_exit interview_ = 6).

**Fig. 2:**
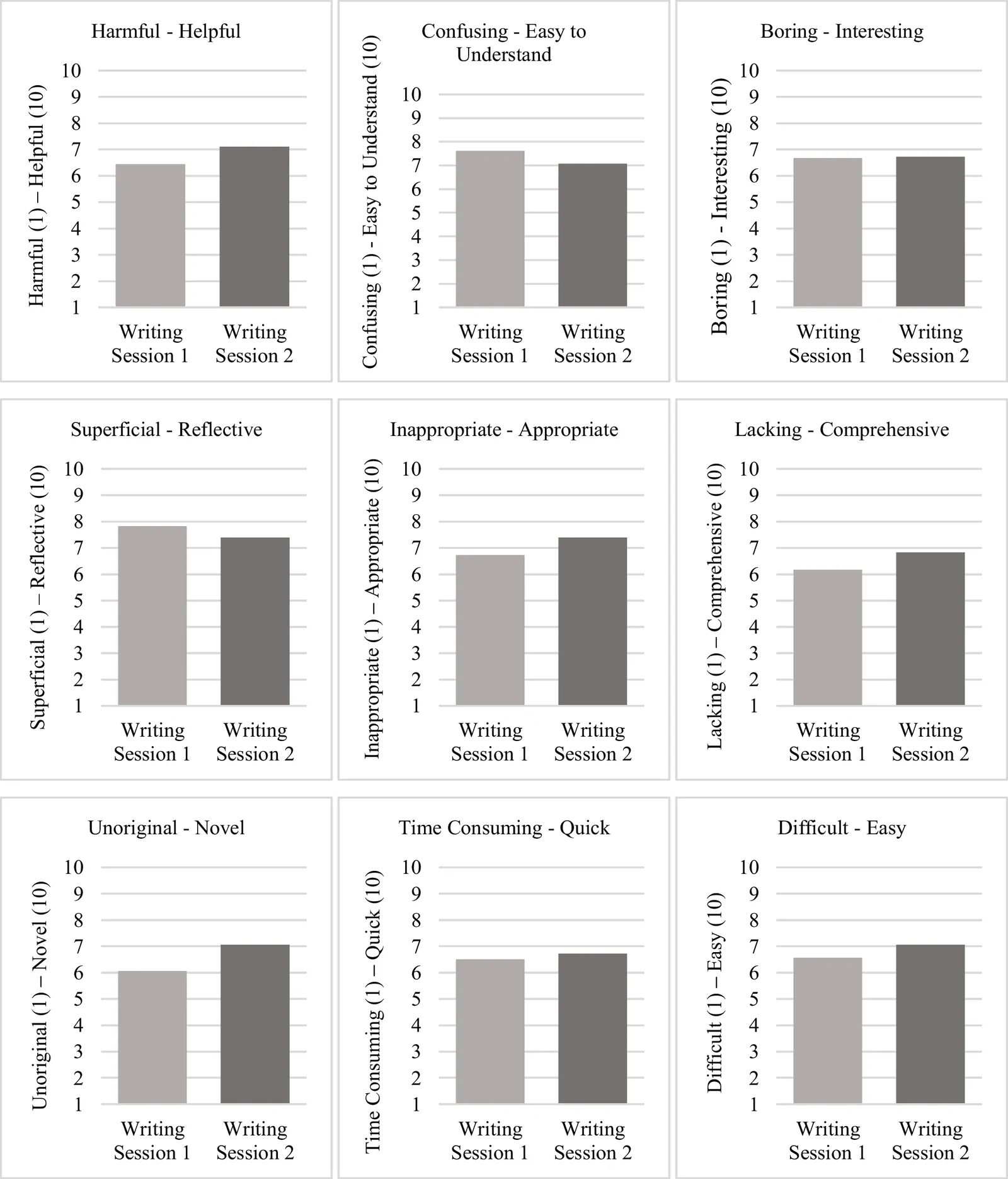
Average scores for quantitative descriptor measure comparing the general prompt (writing session 1) and the writing wrongs prompt (writing session 2; *N* = 18). Note. Higher averages indicate endorsements of positive descriptor words (i.e., helpful, easy to understand, interesting, reflective, appropriate, comprehensive, novel, quick, easy)

When asked how helpful they found the individual writing prompts, more participants found the Writing Wrongs prompt at least *somewhat* helpful (*n* = 15) compared to the general prompt (*n* = 12; see Table 4). Similar trends of positive feedback were observed on a measure where participants rated the sessions using a series of negative and positive word pairs (Figure 2). Notably, participants indicated that the Writing Wrongs prompt was more helpful, appropriate, comprehensive, novel, quick, and easy than the general prompt. Additionally, participants indicated that learning about, learning how to identify, or providing a label for microaggressions was a helpful aspect of the Writing Wrongs prompt in written feedback (Table 5). While all sessions were found to be helpful in some way, the Writing Wrongs session provided help in a way that the general prompt did not.

When asked how much participants liked the prompt’s wording, 94.5% (*n* = 16) of participants indicated that they liked the general prompt at least *somewhat* after Writing Session 1, prior to their exposure to the Writing Wrongs prompt (Table 4). After Writing Session 2, where participants had seen both prompts, 77.8% (*n* = 14) of participants indicated that they liked the Writing Wrongs prompt’s wording at least *somewhat* (Table 4). When asked which prompt participants preferred at the end of Writing Session 2, 77.8% (*n* = 14) of participants indicated a preference for the Writing Wrongs prompt. When asked again after Writing Session 3, 66.7% (*n* = 12) still indicated a preference for the Writing Wrongs prompt. Similar trends were observed in the exit interviews, where most participants’ (*n* = 9) feedback reflected a preference for the Writing Wrongs prompt over the general prompt (Table 6). Three (16.7%) participants recommended changes to the Writing Wrongs prompt, and four (22.2%) recommended changes to the general prompt. Most notably, regarding the Writing Wrongs prompt, participants recommended removing the comparison of microaggressions to “papercuts” as the comparison “didn’t particularly help [them] understand” and could be interpreted as “something that doesn’t hurt and [people are] exaggerating.”

Participants were also asked their opinions on the administration method (i.e., session number, measures used, device used, online format, compensation rate) of this intervention. Participants broadly indicated that they liked the format of the study (Table 6). Most participants liked the 24-h timeframe between each session. About half of the participants requested more sessions, while the other half requested no change. When it came to measures, half of the participants found the measures to be confusing or unhelpful, while four (25%) others found the measures to positively impact their ability to reflect on the writing session. With regard to compensation, 72.2% (*n* = 13) of participants indicated that they felt the compensation was at least *quite a bit* fair.

Most participants (61.1%; *n* = 11) indicated they liked the online format *very much*, and 88.9% (*n* = 16) of participants indicated a preference for online over in-person administration. When considering potential expansion of the intervention, some participants requested additional resources or an option to request feedback on their writing (Table 6). In contrast, some participants preferred the anonymity of the intervention.

All participants who completed exit interviews found the intervention to be necessary due to the helpfulness of the intervention (*n* = 16). One participant reflected:

I think there is value to it. A lot can happen, especially being here at a Predominantly White Institution you go through a lot of stuff and sometimes you may not feel like talking to another person about it…So, being able to write about it you were just able to like kind of get those feelings out and express yourself.

During the exit interview, participants said they would recommend this intervention to a friend by advertising its positive impact (*n* = 16), accessibility (*n* = 4), and minimal time and resource commitment (*n* = 4).

When asked how likely it was that the participant would re-engage with the intervention, 61.1% (*n* = 11) of participants indicated that they at least *somewhat* wanted to re-engage in the intervention. In the exit interviews, all participants indicated that they would re-engage in the intervention in the future (*n* =16). If the intervention were to remain the same, some participants said they would engage in the intervention when they experienced a stressor of any kind (*n* = 10), while others indicated that they would re-engage less frequently (i.e., less than three days in a row; *n* =4). Some participants indicated that they would re-engage in the intervention if it was presented in a different format (i.e., on paper, with feedback, with more specific prompts; *n* = 6).

## Discussion

In the current study, we completed the first six phases of the ADAPT-ITT model [16] to develop Writing Wrongs, an expressive writing intervention, aimed at addressing symptoms resulting from microaggressions experienced by minoritized students at PWIs. We utilized a mixed-methods analysis of data collected through a series of questionnaires and exit interviews with minoritized students currently enrolled at a PWI. In accordance with the study’s aims, we found (a) no attrition and adequate variability for measures, (b) that, across quantitative and qualitative measures, participants perceived Writing Wrongs to be helpful, appropriate, appealing, and necessary, (c) that participants preferred the adapted Writing Wrongs prompt and recommended making the prompt more specific and removing the comparison to “papercuts,” and (d) that some participants requested additional resources or opportunities to receive feedback on their expressive writing entries.

We found that all participants who engaged in the first pre-intervention session completed all three subsequent writing sessions. Notably, this is substantially less attrition than previous studies of online administration of expressive writing [31, 38] and studies assessing the use of online scalable interventions broadly [33]. The lack of attrition demonstrated in the current study is a promising indication of the intervention’s acceptability and retention potential.

We estimated engagement through the measurement of time spent writing. The current study found that participants wrote for less than the instructed 20 min across all writing sessions. Previous literature established the efficacy of such a timeframe [14, 15, 36, 37]; however, qualitative findings for the current study established appropriate engagement for most participants. It is notable that participants were observed to write for longer periods of time in response to the Writing Wrongs prompt, implying increased engagement compared to the general writing prompt. This is further supported by qualitative findings where participants described the Writing Wrongs prompt as easier and less ambiguous. In alignment with recent calls for additional interventions to support coping with discriminatory experiences on campus [8], participants report that Writing Wrongs provided an empowering and purposeful space to process identity-related stressors.

Regarding the content of participants’ expressive writing texts, it is notable that stressors relating to race, ethnicity, or other minoritized identities were present across writing prompts (i.e., the general and Writing Wrongs prompts). Previous literature has established the frequent and pervasive nature of discriminatory experiences, which is consistent with our finding that discussion of stressors related to a minoritized identity occurs across prompts [6, 8, 9]. However, as intended, participants more often wrote about racial/ethnic identity related microaggressions in response to the Writing Wrongs prompt. Our participants reported not writing about a race- or ethnicity-related stressor in response to the general prompt due to normalized discrimination or not knowing that it was an option due to the prompt’s wording. Unfortunately, the sentiment that discussing identity-related stressors is not an option is largely consistent with the lived experience of minoritized students across the literature [6, 8, 9, 55]. Minoritized students report receiving messages at PWIs to assimilate, constrain behaviors, limit ethnic identification, and identify counter-spaces where they can discuss these topics [6, 8, 9, 55]. Furthermore, this finding is consistent with literature which recommends providing specific prompts to individuals from other countries when reviewing trauma exposure as immigration related trauma is not typically endorsed in response to standard trauma inventories [56]. It is valuable to acknowledge that the inclusion of a microaggression-specific prompt provided the opportunity for minoritized students to reflect on such experiences purposefully and may aid in the development of coping skills for the experience of microaggressions. Our study supported the current literature’s timely call [8, 56, 57] for explicitly addressing and naming unique stressors, such as microaggressions, to increase opportunities for coping, education, and intervention through scalable and free resources. Furthermore, this finding also supports the call for additional considerations and adaptations of clinical materials, especially for trauma-informed care, which often fail to consider stressors unique to minoritized individuals [56, 57].

Across all measurements, participants provided primarily positive feedback, indicating that they found the entirety of the intervention (i.e., Writing Sessions 1–3) to be helpful, appropriate, relevant to their lived experience, appealing, and accessible. This finding is further bolstered by all participants indicating that they would recommend the intervention to a friend and re-engage in the intervention in the future.

Participants further reflected that they liked the online format, the timeframe of the sessions, and the compensation. Consistent with the theorized benefits of scalable interventions [28–30], participants indicated that they liked and would advertise that the intervention was accessible, portable, and required minimal time and resources. Additionally, per the first step of the ADAPT-ITT model (Assessment) [16], when asked about the necessity of an intervention for the experience of microaggressions, all participants indicated that an intervention was necessary to aid in coping with these experiences at PWIs. Our findings further bolster current calls for additional scalable coping tools for the experience of discriminatory stress in addition to efforts to address discriminatory acts at the individual level through micro interventions and at the institutional level to eradicate discriminatory acts [8, 58].

When considering the prompts, participants indicated a preference for the adapted Writing Wrongs prompt over the original expressive writing prompt. Consistent with its title, participants reflected that the Writing Wrongs prompt provided an opportunity for them to learn more about microaggressions, the wrongs, and provided a space to utilize the label and reflect on the experience, the writing. Although the feedback was generally positive for the Writing Wrongs prompt, several participants suggested that we add more specificity (i.e., specify timeframe, location) and remove the comparison to “papercuts.” In future iterations, it is recommended that the adapted Writing Wrongs prompt be used across all three writing sessions, in accordance with expressive writing administration protocol [37], and modified to incorporate participant feedback.

When considering the entire intervention, participants provided recommendations such as including opportunities for feedback, additional resources, and the expansion of the project to other minoritized identities (i.e., gender, sexual identity, religious identity). Though these recommendations of feedback and resources would complicate efforts to maintain anonymity, future iterations should consider opportunities for community check-ins, forums, feedback, or resource reminders. Models for post-writing check-ins can be found and potentially adapted from Written Exposure Therapy procedures [59]. Though previous literature has demonstrated the effectiveness of traditional expressive writing methods to other minoritized identities [32, 38], future researchers should consider utilizing the ADAPT-ITT model or the adapted prompt from this study to expand the intervention to other minoritized identities.

### Limitations, Lessons Learned, and Future Directions

Although this study had many strengths, it also had notable limitations. First, our sample, though purposefully small [16], included 18 self-selected participants who predominantly identified as female, all of whom were enrolled at the same university in the southeastern United States. Therefore, the current sample may not be a comprehensive representation of minoritized students at PWIs, and results may not generalize to all minoritized students at PWIs. During recruitment, sample characteristics were monitored to inform advertising efforts in the hopes of recruiting a representative, albeit small, sample. In the future, intentional recruitment planning and identification of key community members to support recruitment may be beneficial in recruiting a more representative sample. Additionally, due to the small sample size and the nature of the theater test, efficacy of Writing Wrongs to reduce distress associated with microaggressions was not established.

The RMAS, a measure used to characterize our sample, demonstrated more modest internal consistency on two subscales, likely resulting from our small sample size and level of endorsements. Though previous literature establishes appropriate psychometrics for this measure in a diverse sample [41], future studies should verify the reliability of these specific subscales (i.e., invisibility, environmental) prior to interpreting their findings. The current study developed measures informed by the ADAPT-ITT model [16] to collect quantitative feedback. The scope of our study was limited; therefore, future studies may consider investigating the measures’ psychometric properties more thoroughly.

In all, the current study highlights the importance of mixed methods to contribute to richer and more nuanced findings in addition to providing the opportunity to amplify the voices of target populations. Future studies may consider additional opportunities to collect qualitative feedback especially for individuals who discontinue or opt out of optional study sessions (e.g., exit interviews).

### Conclusion

The findings of the current study provide support for the perceived necessity and acceptability of Writing Wrongs, an intervention specifically targeting the experience of microaggressions. Participants in the current study also provide valuable recommendations for future improvements of Writing Wrongs. While not the complete solution to eradicating microaggressions, Writing Wrongs is a valuable step towards developing coping skills for the experience of microaggressions through writing. The current study supports progression through the ADAPT-ITT model [16] from the assessment phase into the testing phase, where the fully adapted version of Writing Wrongs will be administered to the target population to test for efficacy. Ultimately, the completion of a clinical trial for Writing Wrongs would result in the first, to our knowledge, adapted, scalable, evidence-based intervention specifically targeting the symptoms resulting from the experience of microaggressions in minoritized students at PWIs. In doing so, Writing Wrongs contributes not only to individual well-being but also to a broader movement towards inclusivity within the educational environment. With support and collaboration from leaders in academia, mental health, and minoritized groups, Writing Wrongs and similar efforts may transform the current educational landscape for the better.

## Supplementary Information

Below is the link to the electronic supplementary material.

Supplementary file1 (PDF 244 KB )

## Data Availability

Data available upon request to the corresponding author.

## Footnotes

1 The African American Acculturation Scale (AAAS-R) [60] was administered to participants who identified as Black or African American; however, poor psychometric properties within this sample resulted in its removal from further investigation. More information on this measure in this study is available upon request.

## Declarations

### Positionality Statement

When the manuscript for this article was drafted, two authors self-identified as White Latina, one author self-identified as Latina, and two authors self-identified as White American.
