# Supplementary material for: Writing Wrongs: Navigating Microaggressions with Adapted Expressive Writing for Minoritized College Students
Source: J Racial Ethn Health Disparities. 2025 Aug 1;13(5):4097–113. doi: 10.1007/s40615-025-02560-0 (PMC13546360; doi:10.1007/s40615-025-02560-0)
Supplement: Supplementary file 1 [file 40615_2025_2560_MOESM1_ESM.pdf]

Table of Contents for Online Supplemental Materials

Description of Expressive Writing Text Analyses 2

Table S1. Measure Administration Schedule 4

Table S2. Time Spent Writing within each session. 5

Table S3. Measures of Acculturation, Microaggressions, and Discrimination. 6

Table S4. Frequency for Everyday Discrimination Scale Responses (*N* = 18) 7

Table S5. Qualitative Themes in response to "What made you decide to write about an ethnically or racially based stressor?" (*N*=4) or "What made you decide not to write about an ethnically or racially based stressor?" (*n*=14) at the end of session 1 (general prompt). 8

Table S6. LIWC-22 Findings based on expressive writing text across sessions (*N*=18)..... 10

Table S7. Qualitative themes of expressive writing texts (*N*=18) 11

Description of Expressive Writing Text Analyses

The LIWC-22 analyzed the content of the writing sessions for each participant and provided variables indicating the tone, emotion, and use of ethnicity related words (See Table S_). Dictionaries, or variables, of interest derived from the LIWC-22 include positive and negative tone, positive, negative, and anxiety related emotional tone, and ethnicity (Boyd et al., 2022; Pennebaker et al., 2015; Tausczik & Pennebaker, 2010). Findings in our sample are consistent with comparable stream-of-consciousness writings described by Boyd et al. (2022). Of note, average negative tone, negative emotion, and anxiety were observed to be lower in texts from Writing Session 2, as compared to the other writing sessions. This trend is consistent with the PANAS, and momentary measure of anxiety findings discussed above. No differences of note were observed for variables relating to positive tone and positive emotions across writing sessions. This implies reduced negativity and anxiety in response to the microaggression prompt, as compared to the general prompt. Additionally, the LIWC-22 variable for ethnicity demonstrated more frequent use of the ethnicity related words in response to the microaggression prompt, as compared to the general prompt. This provides evidence for the efficacy of the microaggression prompt to induce writing about racial/ethnic stressors.

Qualitative analysis considering the expressive writing text of each participant generated three primary themes: racial/ethnic stressor/trauma, other identity stressor, and non-Identity related stressor (See Table S7). These topics arose across writing sessions and prompts. As would be expected, identity-related stressors were more frequently elicited in response to the prompt from Writing Session 2 (n = 18, 100%), as compared to Writing Session 1 (n = 9, 50%) or Writing Session 3 (n = 4, 22%). Such findings are further supported by the LIWC-22 variable for words relating to ethnicity as discussed above. This suggests that the modified prompt had its

intended effect of encouraging participants to reflect on their experiences with microaggressions. Also, as would be expected, in Writing Session 2, non-identity related stressors were not discussed ($n = 0$, 0%). In response to the general prompt in Writing Session 1 ($n = 16$, 89%) and 3 ($n = 17$, 94%), a majority of participants' expressive writing text reflected stressors unrelated to identity. It's important to note that some participants wrote about multiple stressors in one writing session.

Table S1. Measure Administration Schedule

Session	Measures
Eligibility Screener	Demographics (age, gender identity, race, ethnicity, student status)
Pre-Intervention Assessment	Demographics Information Questionnaire<br>Vancouver Index of Acculturation<br>African American Acculturation Scale<br>Racial Microaggression Scale<br>Depression, Anxiety, and Stress Scale<br>Life Events Checklist<br>Posttraumatic Stress Disorder Checklist – DSM-5
Writing Session 1	Pre-Writing PANAS<br>Writing Prompt 1<br>Post-Writing PANAS<br>Assessment of Anxiety and Depression<br>SPRINT<br>Adaptation Evaluation Form 1
Writing Session 2	Pre-Writing PANAS<br>Writing Prompt 2<br>Post-Writing PANAS<br>Assessment of Anxiety and Depression<br>SPRINT<br>Adaptation Evaluation Form 2
Writing Session 3 & Post-Intervention Assessment	Pre-Writing PANAS<br>Writing Prompt 3<br>Post-Writing PANAS<br>Adaptation Evaluation Form 3<br>Depression, Anxiety, and Stress Scale<br>Posttraumatic Stress Disorder Checklist – DSM-5
Exit Interview	Exit Interview Questionnaire

Note. Diagnostic and Statistical Manual of Mental Disorders, Fifth Edition (DSM-5), Positive and Negative Affect Scale (PANAS), Short Post-Traumatic Stress Disorder Rating Interview (SPRINT).

Table S2. Time Spent Writing within each session.

	N	Mean	SD	Min	Median	Max
Time Spent Writing						
Writing Session 1	18	15.0	6.3	6.5	14.8	25.4
Writing Session 2	18	22.6	21.6	4.3	16.0	83.0
Writing Session 3	18	13.5	8.4	0.1	12.9	29.8

Note. All statistics described are in minutes. Time spent writing describes time between first and last click on the expressive writing prompt page.

Table S3. Measures of Acculturation, Microaggressions, and Discrimination.

	n	Measure Range	Observed Range	Mean	SD
VIA					
Dominant	17	1.0 – 9.0	4.0 – 8.4	6.2	1.3
Non-Dominant	17	1.0 – 9.0	4.5 – 9.0	6.6	1.3
RMAS					
Not Belonging/ Being Treated as a Foreigner					
Frequency	18	0.0 – 3.0	0.0 – 3.0	1.2	1.0
Distress [a]	13	0.0 – 3.0	0.0 – 2.0	0.9	0.7
Criminality					
Frequency	18	0.0 – 3.0	0.0 – 1.8	0.5	0.6
Distress [a]	11	0.0 – 3.0	0.5 – 2.7	1.5	0.7
Sexualization					
Frequency	18	0.0 – 3.0	0.0 – 2.7	0.7	0.8
Distress [a]	11	0.0 – 3.0	0.0 – 2.3	1.4	0.7
Low Achieving					
Frequency	18	0.0 – 3.0	0.0 – 2.2	1.1	0.7
Distress [a]	16	0.0 – 3.0	0.5 – 2.9	1.9	0.7
Invisibility					
Frequency	18	0.0 – 3.0	0.0 – 1.3	0.6	0.4
Distress [a]	16	0.0 – 3.0	0.5 – 3.0	1.8	0.7
Environmental Discrimination					
Frequency	18	0.0 – 3.0	0.8 – 3.0	2.1	0.5
Distress [a]	18	0.0 – 3.0	0.0 – 2.8	1.1	0.8

Note. Vancouver Index of Acculturation (VIA) ranges from *Strongly disagree (1)* to *Strongly agree (9)*; Racial Microaggression Scale (RMAS) frequency scale is *Never (0) A little/rarely (1), Sometimes/a moderate amount (2),* and *Often/frequently (3);* RMAS distress scale includes *Not at all (0), A little (1), A moderate level (2),* and *A high level (3)*. Descriptives are based on subscale averages.

[a] Participants only saw this question if they indicated a frequency of at least *A little/rarely (1).*

Table S4. Frequency for Everyday Discrimination Scale Responses (*N* = 18)

	Never<br>*n* (%)	Less than once a year<br>*n* (%)	A few times a year<br>*n* (%)	A few times a month<br>*n* (%)	At least once a week<br>*n* (%)	Almost everyday<br>*n* (%)	Always<br>*n* (%)
"You are treated with less courtesy than other people are."	3 (16.7)	1 (5.6)	10 (55.6)	4 (22.2)	0 (0.0)	0 (0.0)	0 (0.0)
"You are treated with less respect than other people are."	4 (22.2)	4 (22.2)	8 (44.4)	1 (5.6)	1 (5.6)	0 (0.0)	0 (0.0)
"You receive poorer service than other people at restaurants or stores."	7 (38.9)	7 (38.9)	3 (16.7)	1 (5.6)	0 (0.0)	0 (0.0)	0 (0.0)
"People act as if they think you are not smart."	2 (11.1)	7 (38.9)	3 (16.7)	4 (22.2)	1 (5.6)	1 (5.6)	0 (0.0)
"People act as if they are afraid of you."	12 (66.7)	1 (5.6)	4 (22.2)	1 (5.6)	0 (0.0)	0 (0.0)	0 (0.0)
"People act as if they think you are dishonest."	14 (77.8)	3 (16.7)	0 (0.0)	1 (5.6)	0 (0.0)	0 (0.0)	0 (0.0)
"People act as if they're better than you are."	2 (11.1)	5 (27.8)	7 (38.9)	1 (5.6)	2 (11.1)	1 (5.6)	0 (0.0)
"You are called names or insulted."	11 (61.1)	4 (22.2)	2 (11.1)	1 (5.6)	0 (0.0)	0 (0.0)	0 (0.0)
"You are threatened or harassed."	13 (72.2)	3 (16.7)	2 (11.1)	0 (0.0)	0 (0.0)	0 (0.0)	0 (0.0)

Table S5. Qualitative Themes in response to "What made you decide to write about an ethnically or racially based stressor?" (*N*=4) or "What made you decide not to write about an ethnically or racially based stressor?" (*n*=14) at the end of session 1 (general prompt).

Theme	Subtheme	Child Subtheme	Grandchild Subtheme	Description	Writing Session 1<br>*n* (%)
Wrote about an ethnically or racially based				Statements under this code described why participants did write about a racial/ethnic stressor.	4 (22)
	No reason/ Unintentional			Statements under this code imply that discussing racial/ethnic stressors "just happened" or was entwined into the stressor unintentionally. These statements may also indicate that the racial or ethnic stressor is the most stressful experience.	2 (11)
	Identity influenced world view			Statements indicate that the participant's world view/interaction with others consistently relates to or is colored by their ethnicity/race.	1 (6)
	Demand characteristics			Statements under this code implies that the participant wrote about a racial/ethnic stressor because of the study's title or description.	1 (6)
Did not write about an ethnically or racially based stressor				Statements under this code described why participants did not write about a racial/ethnic stressor.	14 (78)

(Cont.)

Theme	Subtheme	Child Subtheme	Grandchild Subtheme	Description	Writing Session 1<br>*n* (%)
	More stressful experience			Statements under this code indicate a stressor that was worse or had a bigger impact on the participant than a stressor related to their identity. Or that an ethnic/race-based stressor didn't come to mind.	14 (78)
		Normalized discrimination		Statements under this code reflect an experience with discrimination that feels normalized and no longer is associated with distress. For example, "I've just grown used to" "never experience personal racial or ethnic stressors that are harmful enough" "get pushed aside."	9 (50)
			Unaware of option	Statements under this code didn't write about racial/ethnic stressors because they didn't think it was an option or the lack of "better phrasing" in the general prompt.	2 (11)
		No microaggressions experienced		Statements under this code indicate not talking about a racial/ethnic stressor because they hadn't faced any.	1 (6)

Note. Participants were only administered separate questions based on whether they indicated that they did or did not write about a racial or ethnically based stressor following Writing Session 1 and the general prompt. Percentages are based on the whole sample (*N* = 18).

Table S6. LIWC-22 Findings based on expressive writing text across sessions (*N*=18)

LIWC-22 Variable Names	Variable Exemplars	Range	Observed Range	Writing Session 1	Writing Session 2	Writing Session 3
				M (SD)	*M* (SD)	*M* (SD)
Positive Tone	good, well, new, love	0 – 100%	0.0 – 5.5%	1.7% (1.2%)	1.9% (1.8%)	1.7% (1.1%)
Negative Tone	bad, wrong, too much, hate	0 – 100%	0.0 – 16.9%	3.6% (2.5%)	1.9% (1.1%)	3.6% (3.6%)
Positive Emotion	good, love, happy, hope	0 – 100%	0.0 – 3.5%	0.7% (0.8%)	0.7% (0.8%)	0.4% (0.5%)
Negative Emotion	bad, hate, hurt, tired	0 – 100%	0.0 – 12.1%	2.8% (2.7%)	1.2% (1.1%)	2.4% (2.1%)
Anxiety	worry, fear, afraid, nervous	0 – 100%	0.0 – 4.6%	1.2% (1.3%)	0.2% (0.3%)	1.1% (0.8%)
Ethnicity	American, French, Chinese, Indian	0 – 100%	0.0 – 4.0%	0.1% (0.4%)	1.0% (1.2%)	0.3% (0.6%)

Note. Linguistic Inquiry and Writing Count (LIWC). Statistics reflect the percentage of total words in participants' expressive writing text related to a variable.

Table S7. Qualitative themes of expressive writing texts (*N*=18)

Theme	Subtheme	Child Subtheme	Grandchild Subtheme	Description	Writing Session 1	Writing Session 2	Writing Session 3
					n (%)	*n* (%)	*n* (%)
Racial/Ethnic stressor/ trauma				The statements under this code include stressors or traumas that were discussed in the writing that were related to a participant's racial or ethnic identity. These include but are not limited to microaggressions and experiences of discrimination, prejudice, and racism.	7 (39)	16 (89)	4 (22)
	Microaggression			Statements under this category include "subtle, innocuous, preconscious, or unconscious degradations, and putdowns" (Pierce 1995, p.218) that occur in daily verbal, nonverbal, or environmental interactions.	1 (6)	15 (83)	2 (11)
		Micro-assaults		Statements in this code include verbal or nonverbal attacks that intend to harm a minoritized individual (Sue et al. 2007).	0 (0)	0 (0)	1 (6)
		Micro-insults		Statements under this code include verbal or non'-'verbal actions that convey rudeness, insensitivity, or are meant to demean based on an individuals minoritized identity (Sue et al., 2007).	0 (0)	13 (72)	1 (6)

(Cont.)

Theme	Subtheme	Child Subtheme	Grandchild Subtheme	Description	Writing Session 1	Writing Session 2	Writing Session 3
					n (%)	*n* (%)	*n* (%)
			Country of origin	The source of such stressor relates to the participants country of origin.	0 (0)	4 (32)	0 (0)
			Race/ Ethnicity	The source of such stressor relates to the participants racial/ethnic background.	0 (0)	11 (61)	1 (6)
		Micro-invalidations		Statements under this code include statements made to exclude, negate, or ignore an individual's thoughts, feelings, or experiences as a minoritized individual (Sue et al., 2007).	1 (6)	7 (39)	1 (6)
			Country of origin	The source of such stressor relates to the participants country of origin.	1 (6)	2 (11)	0 (0)
			Race/ Ethnicity	The source of such stressor relates to the participants racial/ethnic background.	0 (0)	6 (33)	1 (6)
	Structural			Statements relate to stressors that discuss cultural values within a society that become normalized and considered "the way things are" that relate to the treatment of minorized individuals. This may be an explicitly experienced discrimination or internalized sentiments.	3 (17)	4 (22)	1 (6)

(Cont.)

Theme	Subtheme	Child Subtheme	Grandchild Subtheme	Description	Writing Session 1	Writing Session 2	Writing Session 3
					n (%)	*n* (%)	*n* (%)
	Immigration			Statements under this code relate to stressors associated with immigration. This may include the process of immigration and acculturation.	3 (17)	0 (0)	1 (6)
	Institutionalized discriminatory experience			Statements relate to stressors that discuss policies or behaviors within nonspecific organizations or institutions that intend on discriminating against minoritized individuals. This may be an explicitly experienced discrimination or internalized sentiments.	1 (6)	1 (6)	0 (0)
Other identity stressor				Statements under this code involve stressors that relate or are attributed to an aspect of the participants identity that is not encompassed by race/ethnicity.	2 (11)	4 (22)	1 (6)
Non-identity related stressor				The statements under this code include stressors that were discussed in the writing that were not related to a participant's identity.	16 (89)	0 (0)	17 (94)

(Cont.)

Theme	Subtheme	Child Subtheme	Grandchild Subtheme	Description	Writing Session 1	Writing Session 2	Writing Session 3
					n (%)	*n* (%)	*n* (%)
	Interpersonal stress			Statements under this code relate to stressors that stem from or associated with relationships or stressors related to individuals in the participant's social circle.	11 (61)	0 (0)	13 (72)
		Familial stress		Statements under this code relate to stressors related to family members or the family dynamic. This may include but is not limited to stressors in the family life that impact the participant or discord among family members.	6 (33)	0 (0)	9 (50)
		Friendship stress		Statements under this code relate to stressors related to friendships. This may include but is not limited to friendship discord, friendship dissolution, or stressors in the friend's life that impact the participant.	2 (11)	0 (0)	0 (0)
		Partner Stress		Statements under this code relate to stressors related to partnerships. This may include but is not limited to discord within the relationship, dissolution of relationships, or stressors in the partner's life that impact the participant.	2 (11)	0 (0)	2 (11)

(Cont.)

Theme	Subtheme	Child Subtheme	Grandchild Subtheme	Description	Writing Session 1	Writing Session 2	Writing Session 3
					n (%)	*n* (%)	*n* (%)
		Roommate stress		Statements under this code relate to stressors related to roommates. This may include but is not limited to discord with roommates, stressors within the participants living situation, or stressors in the roommate's life that impact the participant.	1 (6)	0 (0)	1 (6)
	Academic			Statements under this code relate to stressors associated with the academic setting. This may include but is not limited to concerns about acceptances, grades, course completion, and more.	4 (22)	0 (0)	6 (33)
	Mental health			Statements under this code relate to stressors associated with mental health difficulties (i.e., anxiety, depression, general mental health concerns).	4 (22)	0 (0)	2 (11)
	Illness or injury			Statements under this code relate to stressors associated with significant illness or injury (i.e., seizures, cancer).	2 (11)	0 (0)	2 (11)

(Cont.)

Theme	Subtheme	Child Subtheme	Grandchild Subtheme	Description	Writing Session 1 *n* (%)	Writing Session 2 *n* (%)	Writing Session 3 *n* (%)
	Sudden death			The statements under this code relate to discussion of a sudden or unexpected death. This code also includes mentions of suicide and suicidal ideation.	2 (11)	0 (0)	2 (11)
		Suicide		The statements under this code relate to suicide. This may include learning about the completed suicide or suicidal ideation of another individual or suicidal ideation experienced by the participant. Such experiences may be described as experiencing suicidal ideation, learning about the ideation of another individual, or being closely involved with an individual who is ideating.	2 (11)	0 (0)	1 (6)
	Accident			The statements under this code relate to transportation or serious accidents at work, home, or during recreational activity.	2 (11)	0 (0)	1 (6)
	Financial			Statements under this code relate to financial stressors.	2 (11)	0 (0)	1 (6)
